# Comparative cytogenetics of some marsupial species (Didelphimorphia, Didelphidae) from the Amazon basin

**DOI:** 10.3897/CompCytogen.v11i4.13962

**Published:** 2017-10-26

**Authors:** Carlos Eduardo Faresin e Silva, Rodrigo Amaral de Andrade, Érica Martinha Silva de Souza, Eduardo Schmidt Eler, Maria Nazareth Ferreira da Silva, Eliana Feldberg

**Affiliations:** 1 Laboratório de Genética Animal, Instituto Nacional de Pesquisas da Amazônia, Campus II, Avenida André Araújo, 2936, Manaus, Amazonas, Brazil; 2 Coleção de Mamíferos, Instituto Nacional de Pesquisas da Amazônia, Campus II, Avenida André Araújo, 2936, Manaus, Amazonas, Brazil

**Keywords:** Marsupials, Amazon basin, C-band, NORs, 18S rDNA, Chromosomal rearrangements

## Abstract

We investigated the karyotype of 18 didelphid species captured at 13 localities in the Brazilian Amazon, after conventional staining, C-banding, Ag-NOR and fluorescent *in situ* hybridization (FISH) using the 18S rDNA probe. Variations were found in the X chromosome, heterochromatin distribution and the 18S rDNA sequence. The main variation observed was in the position of the centromere in the X chromosome of *Caluromys philander* Linnaeus, 1758 and *Marmosa murina* Linnaeus, 1758. For both species, the X chromosome showed a geographical segregation in the pattern of variation between eastern and western Brazil, with a possible contact area in the central Amazon. C-banding on the X chromosome revealed two patterns for the species of *Marmosops* Matschie, 1916, apparently without geographic or specific relationships. The nucleolus organizer region (NOR) of all species was confirmed with the 18S rDNA probe, except on the Y chromosome of *Monodelphis touan* Shaw, 1800. The distribution of this marker varied only in the genus *Marmosa* Gray, 1821 [*M. murina* Thomas, 1905 and *M. demerarae* Thomas, 1905]. Considering that simple NORs are seen as a plesiomorphic character, we conclude that the species *Marmosa* spp. and *Didelphis marsupialis* Linnaeus, 1758 evolved independently to the multiple condition. By increasing the sample, using chromosomal banding, and FISH, we verified that marsupials present intra- and interspecific chromosomal variations, which suggests the occurrence of frequent chromosomal rearrangements in the evolution of this group. This observation contrasts with the chromosomal conservatism expected for didelphids.

## Introduction

In the Americas, subclass Metatheria Huxley, 1880 is represented by the three marsupial orders: Didelphimorphia Gill, 1872, Paucituberculata Ameghino, 1894 and Microbiotheria Ameghino, 1889. The largest of the three American orders is Didelphimorphia, which is represented by the family Didelphidae Gray, 1821, whose species are widely distributed throughout the continent. Didelphidae is the only marsupial group present in Brazil. Together with rodents, they make up an important part of the mammalian fauna of the Amazon region (Voss and Jansa 2009, Wilson and Reeder 2011). Currently, 14 genera and 39 species are recorded in the Amazon basin. Although moderate in terms of species richness, didelphids are abundant in the region (Brandão et al. 2015).

Historically, the first cytogenetic data on American marsupials were recorded by Jordan (1911; cited in Reig et al. 1977), on the spermatogenesis of *Didelphis virginiana* Kerr, 1792. Since then, our knowledge of cytogenetics of American and Australian marsupials has grown significantly. Hayman (1990) reported the karyotype of 178 species of American and Australian marsupials and Svartman (2008) reported 45 karyotypes for American marsupials.

Unlike other mammal orders, such as Rodentia Bowdich, 1821, marsupials show relatively little chromosomal variation (Nagamachi et al. 2015). Chromosomal stability in marsupials was first verified in conventional staining karyotypes that revealed the existence of three main diploid numbers in species from both continents: 14, 18 and 22 chromosomes.

Among all the metatherian families, Macropodidae Gray, 1821 (order Diprotodontia Owen, 1866) is the most diverse in diploid number, varying from 2n=10 to 32. While the American Didelphidae has only the three main diploid numbers, with the most frequent being 2n=14 (Reig et al. 1977, Hayman 1990, Palma and Yates 1996, Carvalho et al. 2002), which has been suggested as the ancestral diploid number of all marsupials (Reig et al. 1977, Westerman et al. 2010). Further comparisons using chromosome banding in American and Australian marsupial species revealed that chromosomal stability is verified not only on the diploid number but also on longitudinal banding patterns that show intense conservation on chromatids (Yonenaga-Yassuda et al. 1982, Rofe and Hayman 1985, Casartelli et al. 1986, Souza et al. 1990, Svartman and Vianna-Morgante 1999).

Limited sampling effort has hampered the estimation of species richness in the Amazon, leaving large gaps in our knowledge of the mammalian fauna (Voss and Emmons 1996, da Silva et al. 2001). Currently, of the 39 species of Amazonian marsupials (Brandão et al. 2015) only 17 have associated cytogenetic data (Nagamachi et al. 2015). However, considering the taxonomic instability of Amazonian marsupials, this representation might not be accurate, since new phylogenetic studies will probably change the current classification of several taxa. Furthermore, the earlier literature often lacks a connection between the karyotype of putative species and the analyzed specimens, making it difficult to verify *a posteriori* the taxonomic identification attributed to a given karyotype.

The number of taxa analyzed to date is also limited, and existing cytogenetic analyses have been usually restricted only to the diploid and fundamental numbers (Nagamachi et al. 2015). New advances in the taxonomic classification of Amazonian marsupials, complementary techniques of cytogenetic analysis (banding, *in situ* hibridization), and added sampling efforts (more specimens, new localities) are necessary to improve current knowledge on the cytogenetics of these animals.

In this study, we analyze the main morphological differences in the sex chromosomes and the C-band pattern of 18 didelphid species from the Brazilian Amazon. In addition, we describe for the first time karyotype for six species (*Monodelphis touan*, Monodelphis aff. adusta, *Monodelphis* sp., *Marmosops impavidus*, *Marmosops bishopi* and *Marmosops pinheiroi*) and discuss these patterns in a broader geographical context, including other regions of Brazil and South America.

## Material and methods

**Figure 1.**
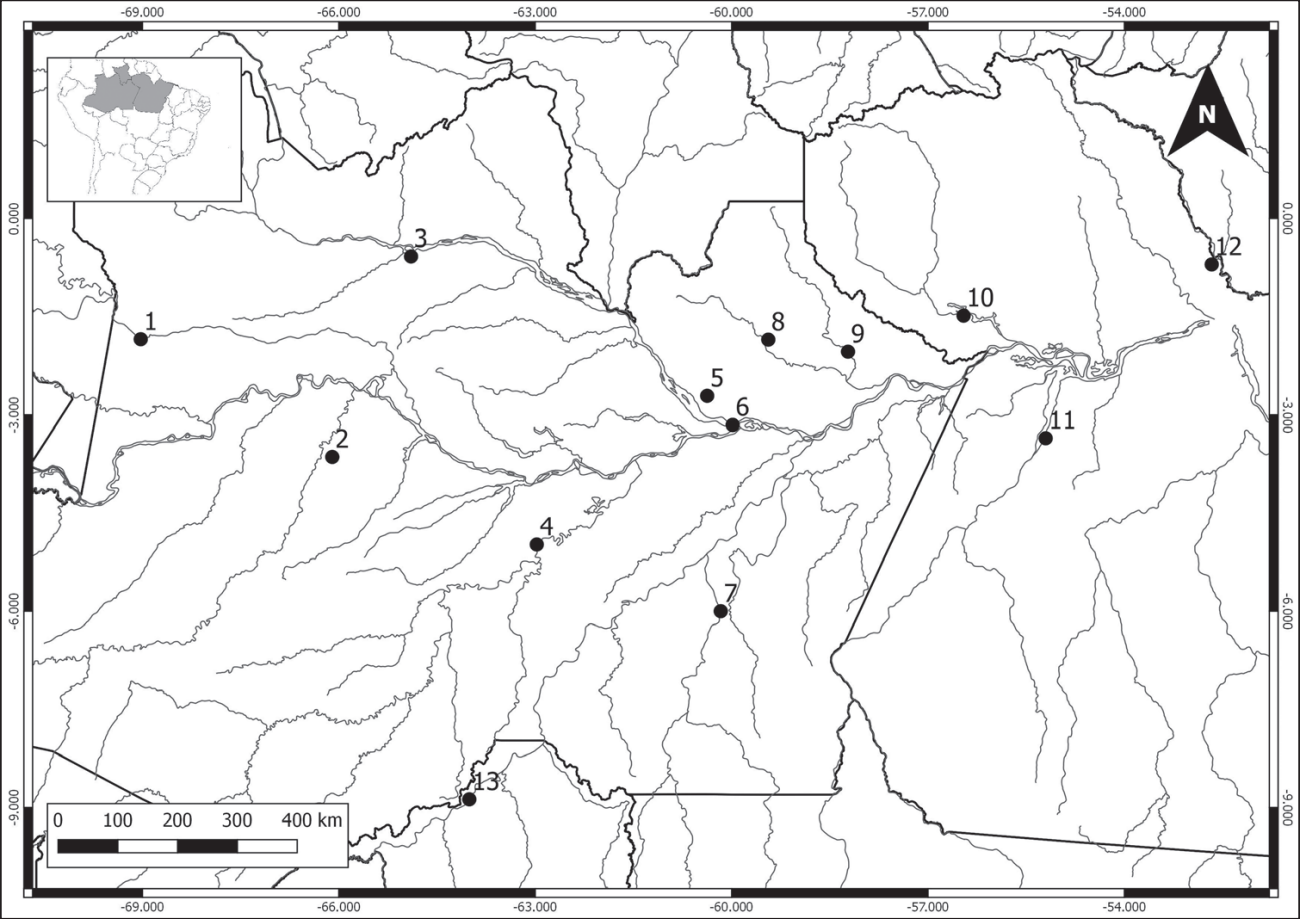
Sampling sites plotted on the Amazon basin map, Amazonas State: **1** Japurá River, Japurá city **2** Juruá River, Juruá city **3** Negro River, Santa Isabel do Rio Negro city **4** Purus River, Tapauá city **5** Cuieiras River, Manaus city **6** Manaus city, urban área: Federal University of Amazonas’s campus (UFAM) and Isaac Sabá Oil Refinery) **7** Aripuanã River, Novo Aripuanã city **8** Uatumã River, Presidente Figueiredo city **9** Jatapú River, São Sebastião do Uatumã city; Pará State: **10** Trombetas River, Oriximiná city **11** Tapajós River, Aveiro and Santarém cities **12** Jari River, Almeirim city; Rondônia State: **13** Madeira River, Porto Velho city. Geographic coordinates at the Table 1.

**Figure 2.**
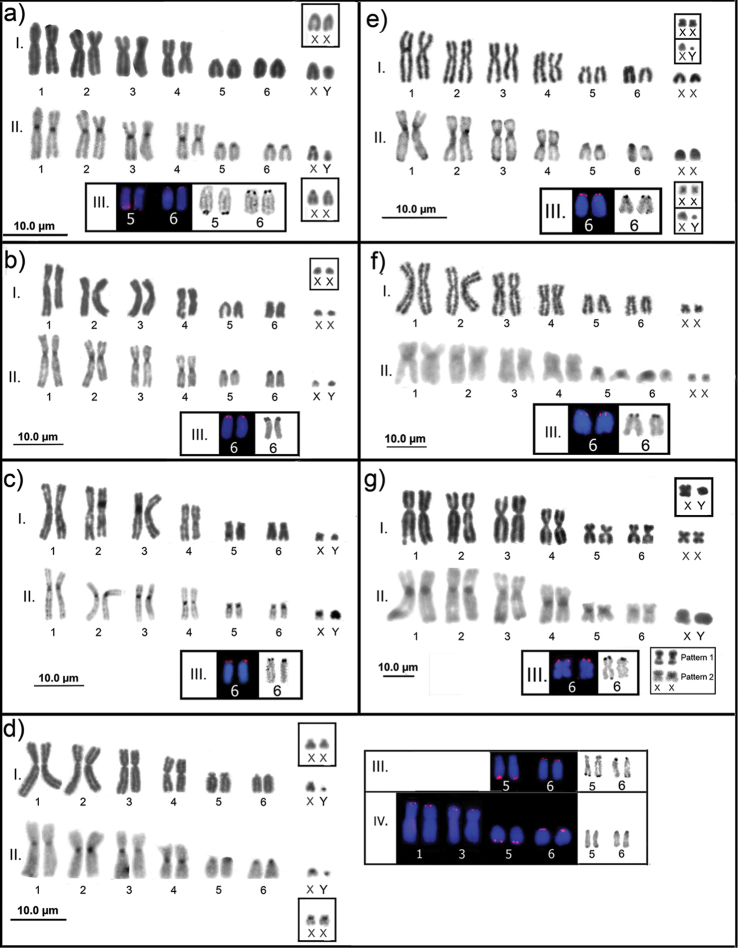
Karyotypes under conventional staining (I), C-band (II), 18S rDNA and Ag-NOR (III), sex chromosomes in the boxes: **a** *Marmosa demerarae* **b** *Metachirus nudicaudatus* **c** *Gracilinanus emiliae* **d** *Marmosa murina*, (IV) variations on the 18S sites found in the individuals from Purus River, Tapauá city, Amazonas State **e** *Caluromys philander* **f** *Caluromys lanatus* **g** *Marmosops pinheiroi*.

**Figure 3.**
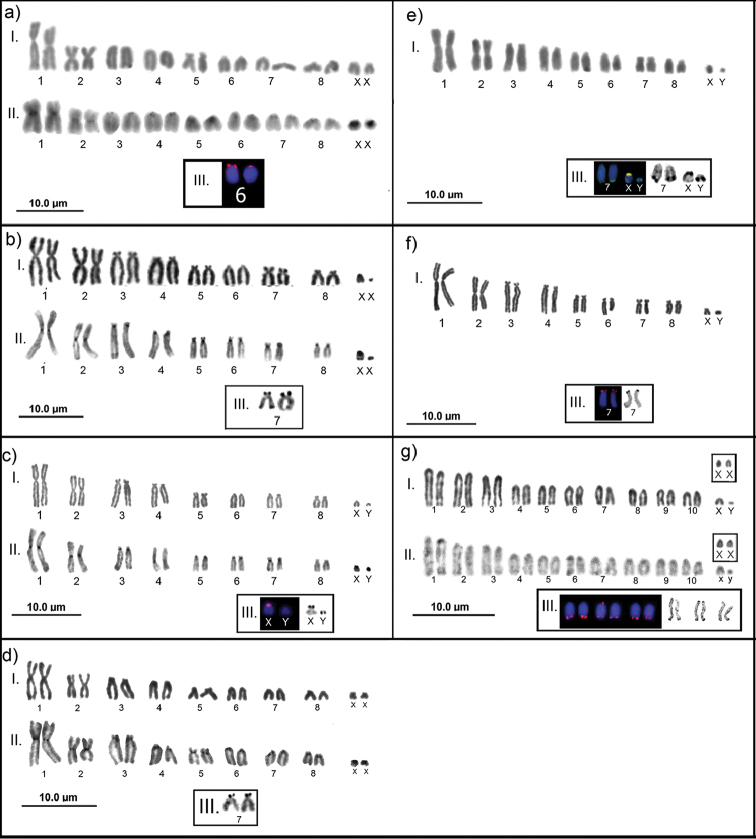
Karyotypes under conventional staining (I), C-band (II), 18S rDNA and Ag-NOR (III), sex chromosomes in the boxes: **a** *Glironia venusta* **b** Monodelphis aff. adusta **c** *Monodelphis touan* **d** *Monodelphis emiliae* **e** *Monodelphis brevicaudata* **f** *Monodelphis* sp. **g** *Didelphis marsupialis*.

We cytogenetically analyzed 111 individuals in 18 species and 8 didelphid genera, collected in 13 localities in the Amazon (Table 1 and Figure 1). Scientific collecting permits were obtained from the Brazilian Institute of the Environment and Renewable Natural Resources (Instituto Brasileiro do Meio Ambiente e Recursos Naturais Renováveis – IBAMA), according to SISBIO license numbers: 02005.000642/03-11 (IBAMA); 02000.002336/2003-93 (IBAMA); 02005.002672/04 (IBAMA); 37585-5 (SISBIO); 37592-4 (SISBIO). The specimens were deposited at the Mammals Collection of the National Institute of Amazonian Research (INPA), Manaus, Brazil. Specimens are indicated by species, sampling sites, genus and collector number, followed by INPA collection number (in parentheses) when available,and their field codes are listed bellow. Karyotyped specimens at the figures: Figure 2: a) *Marmosa demerarae* (RNL 46, boxes: MCA 27); b) *Metachirus nudicaudatus* (SISTAP-M-302; boxes: SISSIS-M-64); c) *Gracilinanus emiliae* (SISTAP-M-243); d) *Marmosa murina* (RNL 69, boxes: CEF 18); e) *Caluromys philander* (SISTAP-M-244, boxes: CAN 34, SISTAP-M-305); f) *Caluromys lanatus* (CTGA-M-701); ; g) *Marmosops pinheiroi* (INPA 5377, boxes: EE 192) (SISTAP-M-278, boxes: EE107, INPA 5408);:. Figure 3: a) *Glironia venusta* (BAC 80); b) Monodelphis aff. adusta (INPA 5388); c) *Monodelphis touan* (INPA 5404); d) *Monodelphis* sp. (CAN 44); e) *Didelphis marsupialis* (EE 249, boxes: EE174)."

**Table 1.** Didelphid species and their respective localities. Species analyzed in the current study were collected at localities 1 to 13, with number of individuals of males (M) and females (F) indicated. Geographic references for the current project were collected in a decimal degree projection using the WGS 84 reference. For literature data we insert converted geographical references where available. Localities with coordinates are presented only the first time they are cited in the table.

Species	Locality	Locality Number	Coordinates†	M	F	Total	Reference
*Caluromys philander*	Trombetas River, Pará State	10	1.48163888889°S, 56.4573333333°W	9	5	14	Present work
	Tapajós River, Pará State	11	3.35486111111°S, 55.2031666667°W	1	1	2	Present work
	Purus River, Amazonas State	4	4.98066666667°S, 62.9770000000°W		1	1	Present work
	Manaus, Amazonas State	6	3.100548°S, 59.974595°W		1	1	Present work
	Aragua, Venezuela	14	–				Reig et al. 1977
	Manaus, Amazonas State	15	3.13333333333°S, 59.9500000000°W				Souza et al. 2013
	Jari, River, Pará State, Brazil	12	0.7000000000°S, 52.6666666667°W		1	1	Souza et al. 2013
	Pernambuco state	16	–				Souza et al. 1990
	São Paulo state	17					Pereira et al. 2008
*Caluromys lanatus*	Japurá River, Amazonas State	1	1.84341666667°S, 69.0264722222°W		1		Present work
	Iquitos, Peru	–	–				Hayman and Martin 1974
	Manaus, Amazonas State	–	–				Casartelli et al. 1986
	Rondônia, Brasil	13	–				Souza et al. 1990
*Marmosa demerarae*	Aripuanã River, Amazonas State	7	6.00000000000°S, 60.1666666667°W	4	4	8	Present work
	Manaus, Amazonas State	6	3.13333333333°S, 59.9500000000°W	7	11	18	Present work
	Cuieiras River, Amazonas State	5	2.70708611111°S, 60.3738388889°W	4	2	6	Present work
	Purus River, Amazonas State	4	0.57725000000°S, 64.8976944444°W	3	4	7	Present work
	Negro River, Amazonas State	3	0.57725000000°S, 64.8976944444°W	1	5	7	Present work
	Tapajós River, Pará State	11	3.35486111111°S, 55.2031666667°W	3	5	9	Present work
	Trombetas River, Pará State	10	1.48163888889°S, 56.4573333333°W	9	5	14	Present work
	Jari River, Pará State	12	0.7000000000°S, 52.6666666667°W	9	2	11	Present work
	Juruá River, Amazonas State	2	3.64151111111°S, 66.1006916667°W		1	1	Present work
	Jatapú River, Amazonas State	9	2.017940°S, 58.203228°W		1	1	Present work
	Jari River, Pará State	12	0.7000000000°S, 52.6666666667°W	1		1	Present work
	Uatumã River, Amazonas State	8	1.84998888889°S, 59.4402000000°W	5	3	9	Present work
	Trombetas River, Pará sate	10	1.48163888889°S, 56.4573333333°W		1	1	Present work
	Negro River, Amazonas State	3	0.57725000000°S, 64.8976944444°W	1	1	2	Present work
	Juruá River	2	3.64151111111°S, 66.1006916667°W		1	1	Present work
*Marmosa murina*	Purus River, Amazonas State	4	0.57725000000°S, 64.8976944444°W	2		2	Present work
	Pernambuco State	16	–				Souza et al. 1990
	Villa Vivencio, Colombia	18	–				Hayman and Martin 1974
	Bolivar, Venezuela	19	–				Reig et al. 1977
	Tartarugalzinho, Amapá State	21					Carvalho et al. 2002
	Loreto, Peru	20	–				Reig et al. 1977
	Vila Rica, Mata Grosso State	22	10°01'S, 51°07'W				Pagnozzi et al. 2002
	UHE Peixe Angical, Tocantins State	23	12°01'30”S, 48°32'21"W				Pereira et al. 2008
	Porto Nacional, Tocantins state	24	10°42'S, 48°25'W				Lima 2004
	Uruaçú, Goiás state	25	14°31'S, 49°08'W				Carvalho et al. 2002
	Colinas do Sul, Goiás state	26	14°09'S, 48°04'W				Carvalho et al. 2002
	UHE Corumbá IV Luziania, Goiás state	27	16°15'09"S, 47°57'01"W				Carvalho et al. 2002
	Pacoti, Ceará state	28	4°13'S, 38°55'W				Pagnozzi et al. 2002
	Reserva Biológica Duas Bocas, Espírito Santo state	29	20°16'S, 40°28'W				Paresque et al. 2004
*Gracilinanus emiliae*	Tapajós River, Pará state	11	3.35486111111°S, 55.2031666667°W	3	1	4	Present work
	Serra da Mesa, Colinas do Sul, Goiás state	26	14°09'S 48°04'W				Carvalho et al. 2002
	UHE Corumbá IV, Luziania,	27	16°15'09"S, 47°57'01"W				Pereira et al. 2008
*Metachirus nudicaudatus*	Trombetas River, Pará state	10	1.48163888889°S, 56.4573333333°W		1	1	Present work
	Jari River, Pará state	12	0.7000000000°S, 52.6666666667°W	1		1	Present work
	Cuieiras River, Amazonas state	5	2.70708611111°S, 60.3738388889°W	1		1	Present work
	Juruá River, Amazonas state	2	3.64151111111°S, 66.1006916667°W		1	1	Present work
	Tapajós River, Pará state	11	3.5486111111°S, 55.2031666667°W	2	2	4	Present work
*Glironia venusta*	Porto Velho, Rondônia State	13	8.87416666667°S, 64.0077777778°W		1	1	Present work
*Monodelphis touan*	Jari River, Pará state	12	0.7000000000°S, 52.6666666667°W	3		3	Present work
*Monodelphis* sp.	Purus River, Amazonas state	4	0.57725000000°S, 64.8976944444°W		1	1	Present work
Monodelphis aff. adusta	Aripuanã River, Amazonas state	7	6.00000000000°S, 60.1666666667°W	1		1	Present work
*Monodelphis emiliae*	Aripuanã River, Amazonas state	7	6.00000000000°S, 60.1666666667°W		1	1	Present work
	Juruá River, Acre state		8°40'S 72°47'W				Patton et al. 2000
*Monodelphis brevicaudata*	Negro River state	3	0.57725000000°S, 64.8976944444°W	1		1	Present work
*Marmosops bishop*	Aripuanã River, Amazonas state	7	6.00000000000°S, 60.1666666667°W	5	6	11	Present work
	Purus River, Amazonas state	4	0.57725000000°S, 64.8976944444°W	2	1	3	Present work
	Negro River, Amazonas state	3	0.57725000000°S, 64.8976944444°W	1		1	Present work
*Marmosops pinheiroi*	Tapajós River, Pará state	11	3.5486111111°S, 55.2031666667°W	4	2	6	Present work
*Marmosops parvidens*	Trombetas River, Pará state	10	1.48163888889°S, 56.4573333333°W	8	1	9	Present work
	Cuieiras River, Amazonas state	5	2.70708611111°S, 60.3738388889°W	3	2	5	Present work
	Jari River, Pará state	12	0.7000000000°S, 52.6666666667°W		2	2	Present work
	Jatapú River, Amazonas state	9	2.017940°S, 58.203228°W	4	3	7	Present work
	La Paz, Bolívia	–	–				Palma and Yates 1996
	Serra da Mesa, Colinas do Sul, Goiás state	26	14°09'S, 48°04'W				Carvalho et al. 2002
	Apiacás, Mato Grosso state		9°34'S, 57°23'W				Pagnozzi et al. 2002
*Marmosops impavidus*	Juruá River, Amazonas state	2	3.64151111111°S, 66.1006916667°W	2	1	3	Present work
*Marmosops pakaraimae*	Japurá River, Amazonas state	1	1.84341666667°S, 69.0264722222°W		1	3	Present work
*Didelphis marsupialis*	Tapajós River, Pará state	11	3.5486111111°S, 55.2031666667°W	1	3	4	Present work
	Trombetas River, Pará state	10	1.48163888889°S, 56.4573333333°W	1	2	3	Present work
	Manaus, Amazonas state	6	3.13333333333°S, 59.9500000000°W	8	4	12	Present work
	Uatumã River, Amazonas stateM	9	2.017940°S, 58.203228°W	1	1	2	Present work
	Cuieiras River, Amazonas state	5	2.70708611111°S, 60.3738388889°W	2	2	4	Present work

All voucher specimens: *Glironia venusta* Thomas, 1912: (BAC 80) – *Caluromys philander* Linnaeus, 1758: Tapajós River (male: SISTAP-M-297; SISTAP-M-305; SISTAP-M-318; SISTAP-M-382; female: SISTAP-M-244); Trombetas River (female: CTGA-M-652); Purus River (female: CAN 34); Manaus (female: MSN 01); (female: BAC 102) – *Caluromys lanatus* Olfers, 1818: Japurá River (female: CTGA-M-701) – *Marmosops* sp. Matschie, 1916: Aripuanã River (female: MCA 3; MCA 7; MCA 8; MCA 26; MCA 31; MCA 35; male: MCA 4; MCA 16; MCA 38; MCA 39); Jari River (female: TAG 3459; RNL 70); Juruá River (male: EE 107; EE 139; female: EE135); Cuieiras River (female: EE 198; EE 211; male: EE 192; EE 201; EE216) – *Marmosops bishopi* Pine, 1981: Negro River (male: SISIS-M-127); Purus River (male: SISPUR-M-135; SISPUR-M-157; SISPUR-M-160; SISPUR-M-164; SISPUR-M-135; CAN 30; CAN 51; female: CAN 48) – *Marmosops pinheiroi* Pine, 1981: Tapajós River (male: SISTAP-M-237; SISTAP-M-278; female: SISTAP-M-268; SISTAP-M-277) – *Marmosops parvidens* Tate, 1931: Trombetas River (male: CTGA-M-501; CTGA-M-516; CTGA-M-531; CTGA-M-532; CTGA-M-551; CTGA-M-555; CTGA-M-581; CTGA-M-600; female: CTGA-M-533) – *Marmosops impavidus* Tschudi, 1845: Purus River (male: SISPUR-M-149) – Marmosops cf. pakaraimae Voss, Lim, Díaz-Nieto et Jansa 2013: Japurá River (male: SISJAP-M-705) – *Marmosa murina* Linnaeus, 1758: Jari River (male: RNL 45); Uatumã River (male: CEF 4; CEF 8; CEF 18; CEF 27; CEF 28; CEF 32; female: CEF 16; CEF 34; CTGA-M-8; CTGA-M-22; CTGA-M-41;), Negro River (male: SISIS-M-57; SISIS-M-63); Trombetas River (female: CTGA-M—519); Purus River (male: CAN 43); Japurá River (male: CTGA-M-708) – *Marmosa murina* Linnaeus, 1758: Aripuanã River (female: MCA12, Japurá River (male: SISJAP-M-764)- *Gracilinanus emiliae* Thomas, 1909: Tapajós River: (male: SISTAP- M-245; SISTAP- M-343; SISTAP- M-344; SISTAP- M-345) – *Marmosa demerarae* Thomas, 1905: Aripuanã River (female: MCA 27; MCA 36; MCA 58; MCA 65; male: MCA 21; MCA59); Jari River (female: RNL 31; RNL 48; male: RNL 30; MCA 32; MCA 46; MCA 49; MCA 58; MCA 61; MCA 64; MCA 66; MCA 67) Juruá River (female: EE136; male: EE 143); Manaus (female: EE 149: EE 150; EE 151; EE 154; EE 158; EE 159; EE 169; EE 222; EE 228; 229; EE 234; male: EE 157; EE 167; EE 170; EE 176; EE 189; EE 194; EE 196; EE 202; EE 215; EE 220; EE 235); Cuieiras River (female: EE 193; EE 219); Tapajós River (female: SISTAP-M-229; SISTAP-M-241; SISTAP-M-321; SISTAP-M-333; SISTAP-M-369; male: SISTAP-M-267; SISTAP-M-279; SISTAP-M-322); Trombetas River (female: CTGA-M-579; CTGA-M-590; CTGA-M-622; CTGA-M-667; CTGA-M-672; male: CTGA-M-535; CTGA-M-539; CTGA-M-557; CTGA-M-558; CTGA-M-572; CTGA-M-573; CTGA-M-578; CTGA-M-580; CTGA-M-613); Negro River (female: SISIS-M-85; SISIS-M-110; SISIS-M-117; SISIS-M-128; male SISIS-M- 86); Purus River (female: SISPUR-M-145; CAN 25; CAN 31; CAN 50: male: SISPUR-M-144; SISPUR-M-147; SISPUR-M-148) – Monodelphis aff. adusta Thomas, 1897: Madeira River (male: MCA 15) – *Monodelphis touan*: Jari River (male: TAG 2731; RNL 68) – *Monodelphis* sp. Burnett, 1830: Purus River: (male: CAN 44) – *Monodelphis emiliae* Thomas, 1912: Aripuanã River (female: MCA 31) – *Metachirus nudicaudatus* Geoffroy et Saint-Hilaire, 1803: Jari: River (RNL 47); Cuieiras River: (female: EE 200); Tapajós River (female: SISTAP-M-230; SISTAP-M-230; male: SISTAP-M-251; SISTAP-M-269); Trombetas River: (female: CTGA-M-655); Jatapú River: (female: CTGA-M-52; CTGA-M-58); Negro River: (female: SISIS-M-64; SISIS-M-78; male: SISIS-M-84; SISIS-M-116); Purus River: (male: CAN 33) – *Didelphis marsupialis* Linnaeus 1758: Jari River: (female: RNL 44; RNL 53; RNL 59; male: RNL 52; RNL 55; RNL 62; RNL 63); Manaus: (female EE 174; EE 197; EE 204; EE 224; EE 227; EE 246; EE 250; EE 155; EE 155; EE 173; EE 183; EE 190; EE 203; EE 205; EE 206; EE 223; EE 232; EE 233; EE 237; EE 247;EE 248; EE249; EE 190); Uatumã River (female: CEF 5; male: CEF 13); Trombetas River (female: CTGA-M-594; CTGA-M-606; male: CTGA-M-607); Purus River (male: SISPUR-M-185); Negro River (male: SISIS-M-73):Tapajós River (female: SISTAP-M-324; SISTAP-M-346; SISTAP–M-347;male: SISTAP-M-243); Japurá River: (male: CTGA-M-732).

The metaphases were obtained from bone marrow by *in vivo* method according to Ford and Harmerton (1956). Each animal received 1mL/100g weight of a 0,0125% colchicine solution for 30 minutes, the cells were exposed for 20 minutes to a 0,075M KCl solution, fixed 3:1 in methanol and acetic acid and stored at -20 °C. The C-band and Nucleolus Organizing Regions (NORs) patterns were determined according to the techniques described by Sumner (1972), and Howell and Black (1980), respectively. Chromosome pairing considered morphology in decreasing order of size and the chromosomes were classified as metacentric (m), submetacentric (sm), subtelocentric (st) and acrocentric (a) according to the ratio of chromosome arms and the position of the centromere, according to Patton (1967). 18S rDNA sequences were mapped by fluorescence *in situ* hybridization (FISH) according to Pinkel et al. (1986), whose probe was obtained by polymerase chain reaction (PCR) using the following primers designed by Gross et al. (2010): 18SF (5’-CCG CTT TGG TGA CTC TTG AT-3’) e 18SR (5’-CCG AGG ACC TCA CTA AAC CA-3’) and labeled with digoxigenin (DIG-Nick translation, ROCHE) or Biotin (Bio-Nick translation, ROCHE), following manufacturer’s instructions.

## Results

Among the 18 species analyzed, 11 showed 2n=14; six 2n=18 and one 2n=22 chromosomes (Table 1).

In the species with 2n=14, we observed a very similar structure among the autosomes. These karyotypes include six autosome pairs (Fig. 2), three large submetacentric pairs, one metacentric pair and two small pairs that varied in morphology in the different species, resulting in differences in the chromosomal formulas and fundamental numbers (FNa). FNa=20, with formula 2m+6sm+4a+XX/XY, was recorded in *Marmosa demerarae* (Fig. 2a-I) and *Metachirus nudicaudatus* Geoffroy an Saint-Hilaire, 1803 (Fig. 2b-I). FNa=22, with formula 2m+6sm+2st+2a+XX/XY, was present in *Gracilinanus emiliae* Thomas, 1909 (Fig. 2c-I), *Marmosa murina* (Fig. 2d-I), *Caluromys philander* (Fig. 2e-I) and *Caluromys lanatus* Olfers, 1818 (Fig. 2f-I). FNa=24, with formula 6m+6sm+XX/XY, was recorded in species of the genus *Marmosops* including *M. bishopi* (Pine, 1981), *M. pinheiroi* Pine, 1981, *M. parvidens* Tate, 1931, *M. impavidus* Tschudi, 1845, and M. cf. pakaraimae Voss, Lim, Díaz-Nieto et Jansa 2013. The five species of *Marmosops* presented similar karyotypic characteristics (Fig. 2g-I – only *M. pinheiroi* shown).

We observed three different morphologies for X chromosome: metacentric in *G. emiliae* and *Marmosops* spp. (Fig. 2c-I and 2g-I); submetacentric in the only female of *C. lanatus* (Fig. 2f-I); and acrocentric in *M. demerarae* and *M. nudicaudatus* (Fig. 2a-I and 2b-I). In *Caluromys philander* and *Marmosa murina*, we observed an intraspecific variation in the structure of the X chromosome, acrocentric and submetacentric, both in specimens from the same and different localities (Fig. 2e-I and 2d-I).

**Figure 4.**
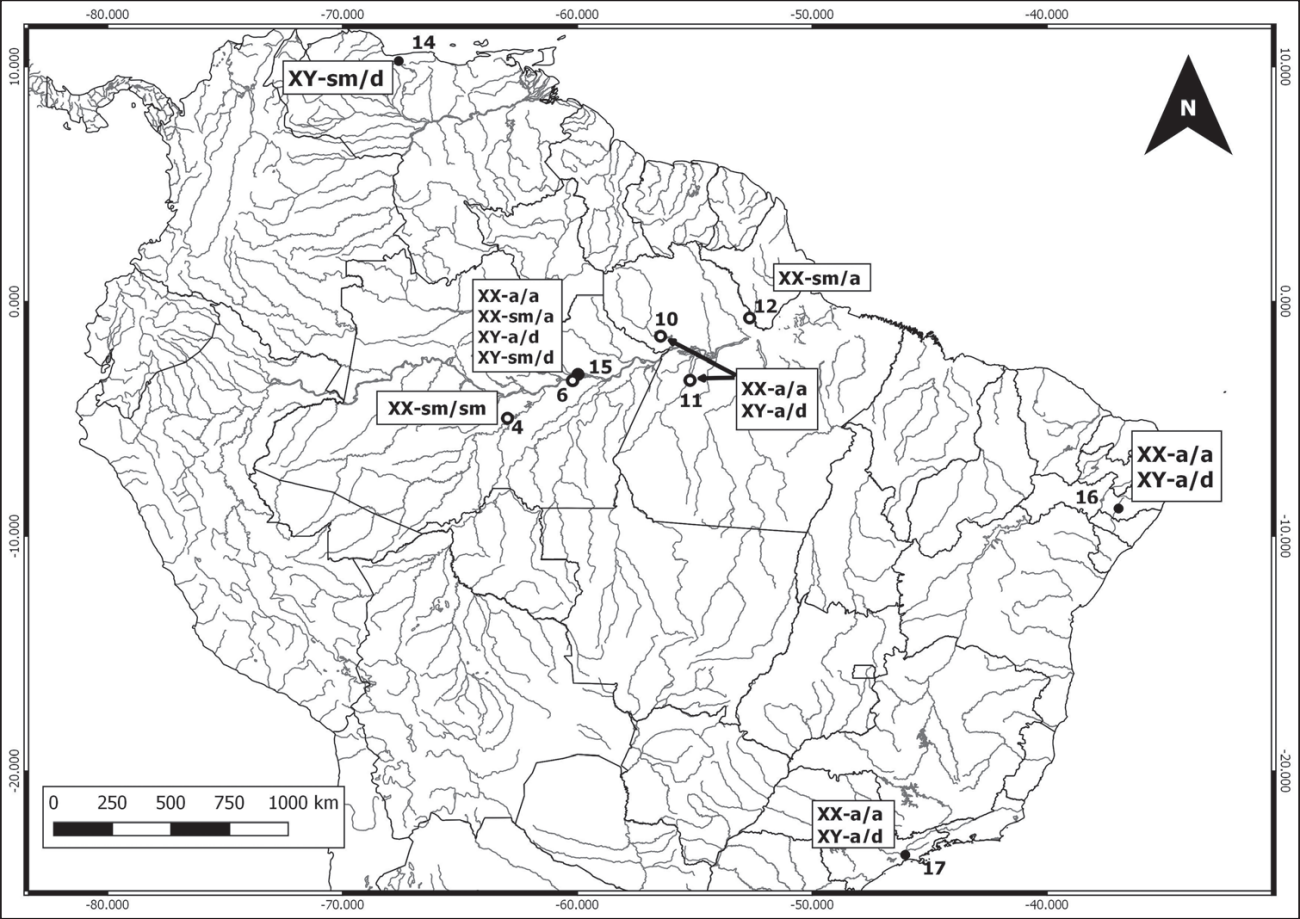
Geographic locations of *Caluromys philander* individuals and its sexual chromosomes morphology data in South America. Literature data represented by empty circles and present work represented by full circles: (● 14) Venezuela, Reig et al. 1977; (○ 4) Purus River; (○ 6) Manaus city, urban área: Federal University of Amazonas’s campus (UFAM); (● 15) Manaus REMAN (Isaac Saba Oil Refinery), present work and Souza et al. 2013; (○ 10) Trombetas River; (○ 11) Tapajós River; (○ 12) Jari River, Souza et al. 2013; (● 16) Pernambuco State, Souza et al. 1990; (● 17) São Paulo State, Svartman and Vianna-Morgante 1999 and Pereira et al. 2008. m = metacentric; sm = submetacentric; a = acrocentric; d = dot-like.

**Figure 5.**
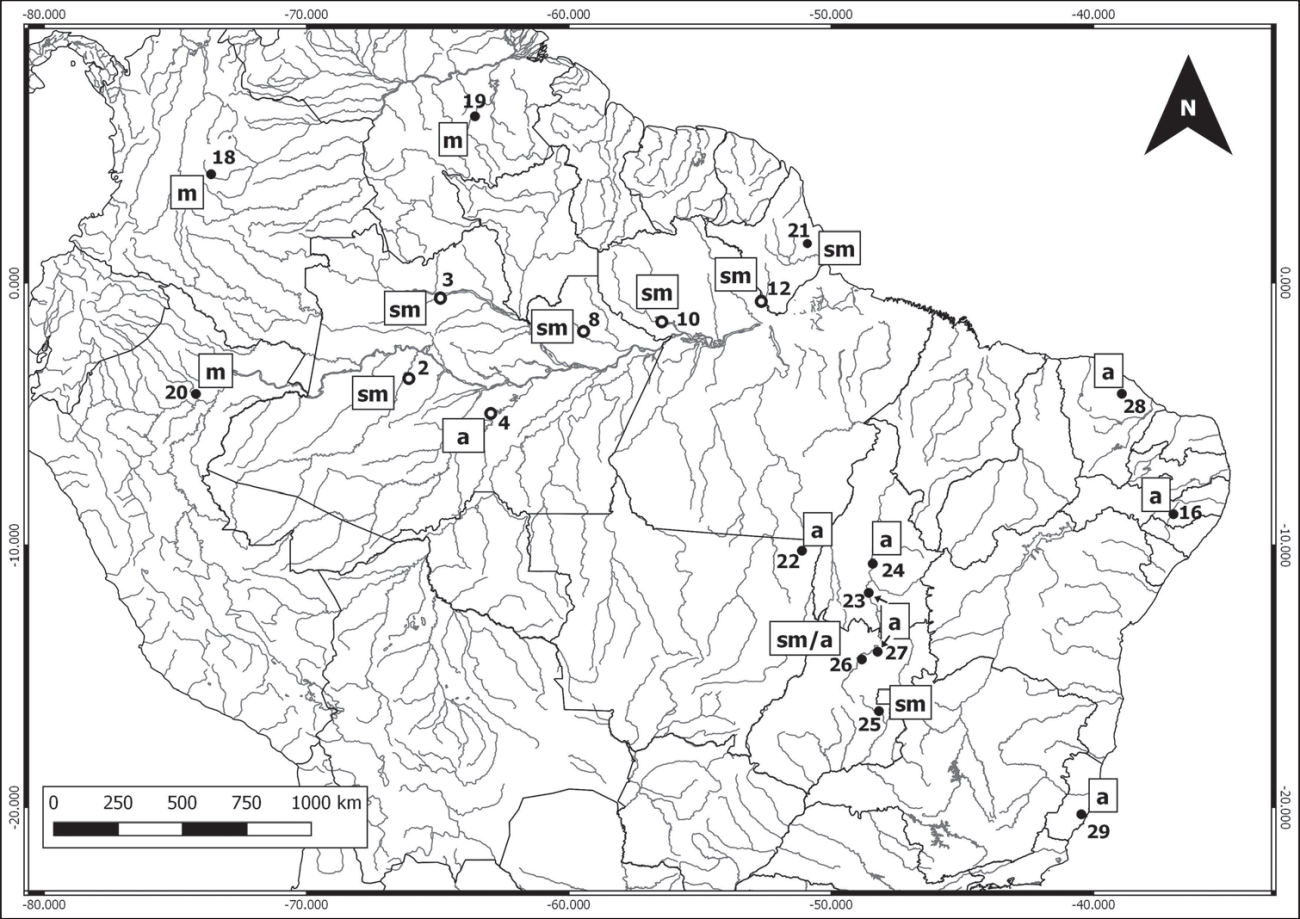
Geographic locations of *Marmosa murina* individuals and its X chromosome morphology data in South America. Literature data represented by empty circles and present work represented by full circles: (● 18) Villa Vivêncio, Colômbia, Hayman and Martin 1974; (● 20) Loreto-Peru, Reig et al. 1977; (● 19) Bolívar, Venezuela, Reig et al. 1977; (○ 3) Negro River; (○ 2) Juruá River; (○ 4) Purus River; (○ 8) Uatumã River; (○ 10) Trombetas River; (○ 12) Jari River; (● 16) Pernambuco State, Souza et al. 1990; (● 21) Tartarugalzinho, Amapá State State, Carvalho et al. 2002; (● 22) Vila Rica Mato Grosso State, Pagnozzi et al. 2002; (● 23) UHE Peixe Angical,TO, Pereira et al. 2008; (● 24) Tocantins State, Lima 2004; (● 25) Uruaçú, Goiás State , Carvalho et al. 2002; (● 26) Colinas do Sul, Goiás state; (● 27) UHE Corumbá IV Luziania, Goiás state Pereira et al. 2008; (● 28) Pacoti, Ceará State, Pagnozzi et al. 2002; (● 29) Espírito Santo State, Paresque et al. 2004. m = metacentric; sm = submetacentric; a = acrocentric.

The bare-tailed woolly opossum (*C. philander*) has X chromosome either acrocentric or submetacentric, with females being either homozygous or heterozygous carriers of the heteromorphic X (Fig. 4). In the murine mouse opossum (*Marmosa murina*), the metacentric or submetacentric X was found in individuals throughout the Brazilian Amazon, except in the Purus River (Fig. 5, locality 4); it was also found in individuals from two localities in central Brazil (Fig. 5, localities 25 and 26). These are situated at the southern limits of the distribution of *M. murina* and both, the submetacentric X and the acrocentric X, are sympatric at locality 26. Furthermore, in the northern Amazon in Colombia, Venezuela and Peru, the X chromosome is metacentric (Fig. 5, localities 18, 19 and 20) (Reig et al. 1977, Carvalho et al. 2002). The acrocentric X was found in the Purus River (Fig. 5, locality 4), and in central, southeastern and northeastern Brazil (Fig. 5, localities 16 and 22-28) (Souza et al. 1990, Palma and Yates 1996, Carvalho et al. 2002).

The Y chromosome was acrocentric in *G. emiliae*, *Marmosops* spp., *M. demerarae* and *M. nudicaudatus* (Fig. 2c, g, a, b), and dot-like in *C. philander* and *M. murina* (Fig. 2e, d).

Among the species with 2n=18 chromosomes, FNa=20 was recorded in *Glironia venusta* Thomas, 1912, with formula 2m+2sm+2st+10a+XX/XY (Fig. 3a-I), FNa=30 was recorded in four species of the genus *Monodelphis* Burnett, 1830: M. aff. adusta Thomas, 1897 (Fig. 3b-I), *M. touan* (Fig. 3c-I), *M. emiliae* Thomas, 1912 (Fig. 3d-I), and *M. brevicaudata* Erxleben, 1777 (Fig. 3e-I) with formula 2m+2sm+8st+2a+XX/XY, and FNa=32 in *Monodelphis* sp. (Fig. 3f-I), with formula 2m+2sm+10st+2a+XX/XY. We observed two X chromosomes morphologies: acrocentric in M. aff. adusta, *M. touan* and *M. brevicaudata* (Fig. 3b, c, e), and submetacentric in *Monodelphis emiliae* and *Monodelphis* sp. (Fig. 3d, f). The Y chromosome was acrocentric in *M. touan* and *Monodelphis* sp., and dot-like in M. aff. adusta and *M. brevicaudata*.

*Didelphis marsupialis* was the only species that presented 2n=22 chromosomes and FNa=20, with formula 20a+XX/XY (Fig. 3g-I), with acrocentric X and Y.

The position of the heterochromatin on the 2n=14 species was centromeric, being conspicuous in *M. demerarae* (Fig. 2a-II), *M. nudicaudatus* (Fig. 2b-II), *G. emiliae* (Fig. 2c-II), *M. murina* (Fig. 2d-II), and *M. pinheiroi* (Fig. 2g-II). *Caluromys philander* and *C. lanatus* exhibited tenuous heterochromatin, with additional telomeric heterochromatin in *C. philander* chromosomes (Fig. 2e-II and 2f-II). The X chromosome in *C. philander* was entirely heterochromatic, except for a distal band in the long arms (Fig. 2e-II); in *M. demerarae* it was also entirely heterochromatic, except for a proximal euchromatic band in the long arms (Fig. 2a-II); in *M. murina* (Fig. 2d-II), *M. nudicaudatus* (Fig. 2b-II) and *G. emiliae* the heterochromatin was centromeric (Fig. 2c-II).

Two C-band patterns were present in the X chromosome for species of *Marmosops*. In pattern 1, X was entirely heterochromatic except for a proximal band in the long arms (Fig. 2g – box); in pattern 2, the heterochromatin was in the short arms and the centromere (Fig. 2g – box). Both patterns were present in *M. parvidens* and *M. bishopi*, while only pattern 1 was observed in M. cf. pakaraimae, *M. impavidus* and *M. pinheiroi* (Table 2). The Y chromosome was entirely heterochromatic in all species.

**Table 2.** Comparative cytogenetic data of the didelphid species analyzed in the present study and those from the literature. In Locality, numbers indicate sampling sites as in the maps of Figures 1, 4 and 5. Karyotypic data: 2n = diploid number; FNa = autosomal arm number; NOR = Nucleolar Organizer Region; p = short arm; q = long arm. Letters identify the X chromosome morphology: m = metacentric; sm = submetacentric; a = acrocentric; d = dot-like. X chromosome C-Band patterns are identified by A = Centromeric heterochromatin; B = Totally heterochromatic except for a terminal euchromatic band; C = Totally heterochromatic except for an interstitial euchromatic band; D = short arm and centromere totally heterochromatic.

Species	Locality number	2n	FNa	NORs Pair/arm	18S rDNA	X/Y	X chromosome C–band	Source
*Caluromys philander*	10; 11; 15; 16	14	22	6p	6p	a/d	B	Souza et al. 1990; Souza et al. 2013; Present work
	4; 6; 14; 15; 17	14	22	6p	6p	sm/d	B	São Paulo State, Svartman and Vianna–Morgante 1999, Pereira et al. 2008, Souza et al. 2013, Present work
	12	14	22	6p	6p	sm/a	B	Souza et al. 2013
*Caluromys lanatus*	1	14	22	6p	6p	sm/–	A.	Present work
*Marmosa murina*	2; 3; 8; 10; 12; 18; 19; 20; 25; 26	14	22	5q;6p	5q;6p	(m)sm/ d	A	Hayman and Martin 1974, Reig et al. 1977, Pereira et al. 2008, Carvalho et al. 2002, Present work
	16; 22; 25; 24; 26; 27; 28; 29	14	22	5q;6p	5q;6p	a/ d	A	Carvalho et al. 2002, Pagnozzi et al. 2002, Lima 2004, Paresque et al. 2004, Pereira et al. 2008
	4	14	22	5q;6p	1p; 3p; 5q; 6p	a/d	A	Present work
*Marmosa demerarae*	2; 3; 4; 5; 6; 7; 9; 10; 11; 12 25, 26	14	20	5q; 6p	5q;6p	a/a	C	Carvalho et al. 2002, Present work
	La Paz, Bolívia	14	20	–	–	a/a	–	Palma and Yates 1996
	16	14	24	5q; 6p		a/a		Souza et al. 1990;
	–	14	24	5pq; 6p	5pq; 6p	a/a		Svartman and Vianna Morgante 2003
	Rio Grande do Sul	14	24	5pq; 6p	–	a/a		Carvalho et al. 2002
*Marmosops bishopi*	4; 7;	14	24	6p		m/a	C; D	Present work
	3	14	24	6p		m/a	C	Present work
*Marmosops pinheiroi*	11	14	24	6p	6p	m/a	C	Present work
*Marmosops parvidens*	5; 10; 12	14	24	6p		m/a	C; D	Present work
	9	14	24	6p		m/a	D	Present work
*Marmosops impavidus*	2	14	24	6p		m/a	C	Present work
*Marmosops pakaraimae*	1	14	24	6p		m/a	C	Present work
*Gracilinanus emiliae*	11; 25; 26	14	22	6p	6p	m/a	A	Carvalho et al. 2002, Present work
*Metachirus nudicaudatus*	2; 5 10; 11; 12	14	20	6p	6p	a/a	A	Present work
*Glironia venusta*	13	18	20	6p	6p	a/–	A	Fantin e da Silva 2011, Present work
*Monodelphis touan*	12	18	28	Xp	Xp	a/a	A	Present work
*Monodelphis sp.*	4	18	32	7p	7p	sm/a	–	Present work
*Monodelphis* aff.*adusta*	7	18	30	7p		a/d	A	Present work
*Monodelphis emiliae*	7, Juruá River, Acre	18	30	7p		sm/–	A	Patton et al. 2000, Present work
*Monodelphis brevicaudata*	3;	18	30	7q, Xp, Yq	7q, Xp, Yq	sm/a	–	Present work
	Roraima and Pará states	18	30	Xp		a/d		Carvalho et al. 2002
*Didelphis marsupialis*	5; 6; 9; 10; 11	22	20	5q;7pq;8q	5q;7pq;8q	a/a	A	Present work

In the species with 2n=18 chromosomes, the heterochromatin was centromeric in *G. venusta* (Fig. 3a-II), M. aff. adusta (Fig. 3b-II), *M. touan* (Fig. 3c-II) and *M. emiliae* (Fig. 3d-II). The Y chromosome was entirely heterochromatic in *M. adusta* (Fig. 3b-II) and *M. touan* (3c-II). It was not possible to determine the C-band pattern in *Monodelphis* sp. and *M. brevicaudata*.

NORs confirmed by FISH using the 18S rDNA probe were present in the short arms of pair 6 in all 2n=14 species and *G. venusta* (2n=18). In *M. demerarae* and *M. murina* sites were also detected in the terminal position of the long arms of pair 5 (Fig. 2, a-III e d-III). In *M. emiliae* (2n=18) the NOR was positioned on the short arms of pair 7 (Fig. 3d-III), and in *M. touan* in the X and Y chromosomes, although no 18S site was detected in Y (Fig. 3c-III). Only *Monodelphis brevicaudata* exhibited multiple NORs (Fig. 3e-III), whose sites were in the terminal region of the long arms of pair 7 and the short arms of X and Y.

In *D. marsupialis*, both the 18S rDNA probe and silver were detected in three chromosome pairs. In two pairs, the sites were located in the terminal region of the long arms, while in one pair they were bitelomeric (Fig. 3g-III). However, in regards to activity, there was a variation of four to eight markings.

## Discussion

In the last decade, advances in systematic and taxonomic studies of the family Didelphidae introduced changes in the taxonomy and nomenclature of several of its taxa (Jansa and Voss 2000, Voss and Jansa 2003, Voss and Jansa 2009, Rossi et al. 2010, Gutiérrez et al. 2010). We used the phylogenetic tree of Jansa and Voss (2014) to map the cytogenetic data of the 18 species we have analysed in order to gain an understanding of chromosome evolution in the group. This work represents the most updated phylogeny of the intergeneric relationships of didelphid marsupials, making our interpretation of the cytogenetic data more integrative than a mere consideration of chromosomal data, and more accurate in light of an independently generated phylogenetic hypothesis.

The autosome structure observed here corroborates karyotypic conservation in the diploid number and chromosomal formula (NFa) as previously described in the didelphid species *Didelphis marsupialis, Marmosa demerarae, Metachirus nudicaudatus, Monodelphis touan* (previously named *M. brevicaudata*), Monodelphis aff. adusta (previously named as M. cf. emiliae) and for species of *Marmosops* (Reig et al. 1977, Yonenaga-Yassuda et al. 1982, Casartelli et al. 1986, Hayman 1990, Souza et al. 1990, Palma and Yates 1996, Svartman and Vianna-Morgante 1998, 1999, Carvalho et al. 2002).

Although didelphids are generally considered to have a conserved karyotype, by comparing the karyotypes among different genera, it was possible to associate them with certain species due to the presence of diagnostic characters. For example, *M. demerarae* and *M. murina* differ in their FNa, morphology, and sex chromosome size. In species of the genus *Monodelphis*, morphological variation in chromosomes was restricted to pair 6, which grants an FNa varying between 30 (as observed in M. aff. adusta, *M. touan* and, *M. brevicaudata*) and 32 arms (*Monodelphis* sp.). However, the same does not occur for the genus *Marmosops*, in which the five species analysed, present a very similar chromosome macrostructure.

The genus *Marmosa* has a complex taxonomy and recently underwent great taxonomic changes, with all species of *Micoureus*, formerly treated as a separate genus, now considered as a subgenus of *Marmosa*. Considering the taxonomic instability in Didelphidae, with individuals being reclassified, and some complex of species being divided into two or more valid taxa, even purportedly karyotyped species may in fact have their karyotypes still unknown. Thus, our knowledge as to how many and which species among didelphids were karyotyped remains unstable. A revision of the literature for species with reported karyotypes is required.

### X chromosome variations

Souza et al. (2013) observed different forms of the X chromosome in *Caluromys philander*, and our data contribute to show their wide geographic distributions. The acrocentric X are found in northeastern and southeastern Brazil (Fig. 4, localities 16 and 17), as well as in central (Fig. 4, locality 6) and eastern Amazon (Fig. 4, localities 10, 11 and 12). While submetacentric form is located in Venezuela (Fig. 4, locality 14) and areas in the western, central and eastern Amazon (Fig. 4, localities 4, 6, 12 and 15) (Reig et al. 1977, Svartman and Vianna-Morgante 1999, Pereira et al. 2008). Interestingly, both homozygote and heterozygote females were recorded in central Amazonia near Manaus (Fig. 4, locality 6). It is not clear how often this condition is found in natural populations. Indeed, so far, the few heterozygous records for X might be related to the low number of captured and cytogenetically analyzed individuals.

Apparently, there is a likely geographical structure in the distribution of the morphological forms of the X chromosome in *Marmosa murina*, with the metacentric X so far found in the northern and western parts of its distribution, the submetacentric X prevailing in the Amazon basin of Brazil and the acrocentric forms prevailing in the other known localities in central and eastern Brazil (Fig. 5). According to Brito et al (2015), this species is currently under revision and is likely to be split into three species. It remains to be seen if there will be a correspondence between those species and the karyotypic forms depicted here.

Among the Amazonian marsupials analyzed here, the variation in centromere position and heterochromatin patterns of the X chromosome is noteworthy. Souza et al. (2013) suggested that pericentric inversions in the X chromosome of *Caluromys philander* altered its morphology, and our results support their findings. In contrast, in *M. murina*, the different morphologies (m, sm, and a) of chromosome X might be due to centromeric shift without the presence of rearrangements. Such reorganization was already observed in other mammals and might be related to three main regions of the chromosome: subtelomeric, proximal and in the boundary between heterochromatin and euchromatin (Rocchi et al. 2012, Burrack and Berman 2012).

### Heterochromatin distribution

We observed chromosomal conservatism in the heterochromatin pattern in eight didelphid species: (*C. lanatus*, *G. venusta*, *D. marsupialis*, *M. touan*, M. aff. adusta, *M. emiliae*, *G. emiliae* and *M. nudicaudatus*). *C. philander* presented heterochromatic pattern different from the heterochromatic distribution reported in the literature for this species (Souza et al. 1990, Souza et al. 2013). In *Marmosops* spp., the C-band patterns of the X chromosome are widespread throughout the Amazon basin, but are found in sympatry in the area between the confluences of the Negro-Purus and the Trombetas-Tapajós Rivers, forming pattern 1 to the west and pattern 2 to the east (Table 2). It remains to be seen if there is a correspondence between these patterns with possible cryptic species to be uncovered by broader molecular systematics and morphological studies of these taxa.

Thus, heterochromatin distribution patterns can serve as a cytotaxonomic character, as well as shedding light on chromosomal evolution and regulation of gene expression. However, our results demonstrate that, except for *Marmosops* spp., the other species under study presented little heterochromatin intraspecific variation, including the X chromosome. Thus, this character alone does not allow for distinguishing among didelphid populations, although heterochromatin distribution may be an effective character for distinguishing between certain species pairs. This is the case for *M. demerarae* and *M. murina*, with the former presenting larger centromeric heterochromatic blocks than the latter, and between *C. philander* and *C. lanatus*, both with 2n=14 and NF=24, but with distinct heterochromatic patterns.

### Nucleolus organizer regions (NORs) and their evolution

The NOR in Didelphidae can be simple or multiple. According to Hsu et al. (1975), the single NOR would be an ancestral character in mammals, with subsequent rearrangements leading to multiple NORs in derived groups. The presence of NOR in sex chromosomes also could be considered a derived character since originally it was present in autosomes and ended up in the X chromosome due to rearrangements such as translocation or transposition. The NOR in *Glironia*, *Monodelphis, Caluromys, Gracilinanus*, and *Marmosops* is simple. Thus, these genera have a plesiomorphic condition for this character. Conversely, the species of *Didelphis*, *Marmosa* and *Philander* have the derived condition of multiple NORs (Yonenaga-Yassuda et al. 1982, Svartman and Vianna-Morgante 2003).

According to the literature, in *Monodelphis* there are NOR sites on pair 7 and on the X chromosome of Monodelphis aff. adusta and *Monodelphis* sp. (Svartman and Vianna-Morgante 1999, Merry et al. 1983, Carvalho et al. 2002). In *M. touan* and *M. brevicaudata* there are simple NORs on the X and Y chromosomes, a condition previously identified in *Monodelphis domestica* Wagner, 1842 (Merry et al. 1983, Pathak et al. 1993). Hsu et al. (1975) reported ribosomal genes in mammal sex chromosomes of the bat species *Carollia castanea*. These authors emphasize that NOR in the X chromosome can generate problems with dosage compensation in mammals.

In the Y chromosome of *M. touan*, FISH did not confirm the marking. This situation was verified in other organisms, where precipitation in the heterochromatic regions took place but could lead to an erroneous interpretation of the distribution of this marker (Schneider et al. 2012). Thus, the marking observed (Fig. 3c III) was not a ribosomal site but a heterochromatic block with silver affinity.

When mapping the NOR character on the phylogenetic tree of Jansa and Voss (2014, fig. 01) (not shown here), we verified that multiple NORs are distributed in two distinct lineages: the first in species of the genus *Marmosa* and the second in species with 22 chromosomes of the genera *Didelphis* and *Philander* Brisson, 1762. The mapping of the simple condition onto the phylogenetic tree depicts a wide distribution for this character, present at the base of the tree (*Caluromys philander, C. lanatus, Glironia venusta*) and in at least one or more species of the remaining major clades (*Gracilinanus emiliae, Marmosops* spp., *Metachirus nudicaudatus, Monodelphis touan, Monodelphis kunsi, and Monodelphis dimidiata*) (Souza et al. 1990, Palma and Yates 1996, Carvalho et al. 2002, Svartman and Vianna-Morgante 2003, Pereira et al. 2008, Souza et al. 2013). This distribution of NOR character on the didelphid phylogeny is thus congruent with the hypothesis advanced by Hsu et al. (1975) that the single NOR is an ancestral state.

When mapping the NOR character on the phylogenetic tree of Pavan et al. (2014) for the genus *Monodelphis*, we verified that *M. emiliae, Monodelphis* sp. and Monodelphis aff. adusta seem to have retained the plesiomorphic condition of a simple NOR. Conversely, this condition became variable in *M. domestica* and in the *M. brevicaudata* species complex, which in addition to the NOR identified in the autosomal pair 7, also presents NORs in both chromosomes of the sex pair, indicating a duplication of this site.

In *M. murina*, intraspecific geographic variation in NORs were detected. Specimens from the Purus River have multiple NORs, those collected in the state of Goiás have simple NOR in the short arms of pair 6 (Palma and Yates 1996, Carvalho et al. 2002) and those from the state of Pernambuco present additional markings in the long arms of pairs 3 and 5 (Souza et al. 1990). Furthermore, both specimens from the Purus River differed from the others regarding sex chromosomes.

Our results indicate geographic variation in NORs for *M. demerarae*. Amazonian specimens analysed did not present ribosomal cistrons in the short arms of the fifth pair, as recorded for specimens from the Atlantic forest in the Rio Grande do Sul and São Paulo states of southern Brazil (Carvalho et al 2002, Svartman and Vianna-Morgante 2003, Svartman 2008). Several studies have shown that considerable genetic variation exists among referred populations of this taxon (Voss and Jansa 2003, Dias et al. 2010, Gutiérrez et al. 2010). Therefore, several nominal taxa previously considered synonyms are now treated as valid species. Currently, *M. demerarae* is considered to occur in south to northern and central Brazil, and to southern Bahia (Gardner 2008, Dias et al. 2010) and *Marmosa paraguayana* Tate 1931 occurs from northern border of Espírito Santo state, south to Rio Grande do Sul, and east to Misiones (Argentina), and eastern Paraguay (Gardner 2008). However, some authors consider it to go as far north as Pernambuco state in northeastern Brazil (Voss and Jansa 2003). Thus, considering the geographic distribution of this taxon, the 18S rDNA data presented for locations in northern and eastern Brazil possibly belong to specimens of *M. paraguayana*. As such, this character would have a cytotaxonomic value, and rearrangements involving the ribosomal sites could be related to speciation events related to this sister-species pair (Gutiérrez et al. 2010).

In *Didelphis marsupialis* from several Amazonian sites, only NOR activity varied, as was already reported in specimens from the Atlantic forest (Yonenaga-Yassuda et al. 1982, Svartman and Vianna-Morgante 2003).

## Conclusion

Dutrillaux (1979) suggested that a small sample size would be inadequate for the knowledge of species karyotypes. Heeding this admonition, we used a relatively large number of individuals for each species analysed to uncover a range of variations that most likely would not have been detected had we used fewer individuals per species. The use of integrative analyses and new methodologies, such as taxonomy, phylogeny, and molecular cytogenetics could improve our understanding of the significance of these chromosomic variations. However, for the Amazon region, a significant limitation for cytogenetic studies is still the restricted collection effort, the vast geographical extent of the region and the difficulty of access to remote areas.

The cytogenetic data presented here shows that didelphid marsupial karyotypes present intraspecific variation in the morphology of sex chromosomes and in chromosomic markers (C-band and NOR) and present some geographic variation in the distribution of these features for several species. Furthermore, there are many areas in the Amazon, including the transition zone between the Amazon and the Cerrado biomes, which do not have cytogenetic records for any didelphid species. This situation seriously undermines our understanding of the significance of the recorded variation, whether it is part of a continuous gradient, or whether it represents intraspecific gradations, or whether it is related to new lineages or cryptic species still uncovered. Thereby, despite the chromosomal stability related to diploid numbers and chromosomal formula in marsupials across continents, didelphids present some intra- and interspecific chromosomal variations, probably related to frequent chromosomal rearrangements. Additional systematic sampling and analyses will be required for a better understanding of the karyotypic evolution of this group.

## Supplementary materials

Supplementary material 1 Voucher specimens Data type: Microsoft Word Document (.docx) Explanation note: All analyzed specimens were deposited at Mammals Collection in the Instituto Nacional de Pesquisas da Amazônia (INPA); specimens are indicated by species, sampling sites, genus and collector number, followed by INPA collection number (in parentheses) when available. This dataset is made available under the Open Database License (http://opendatacommons.org/licenses/odbl/1.0/). The Open Database License (ODbL) is a license agreement intended to allow users to freely share, modify, and use this Dataset while maintaining this same freedom for others, provided that the original source and author(s) are credited.Carlos Eduardo Faresin e Silva, Rodrigo Amaral de Andrade, Érica Martinha Silva de Souza, Eduardo Schmidt Eler, Maria Nazareth Ferreira da Silva, Eliana Feldberg
