# Supplementary material for: Comparative cytogenetics of some marsupial species (Didelphimorphia, Didelphidae) from the Amazon basin
Source: Comp Cytogenet. 2017 Oct 26;11(4):703–25. doi: 10.3897/CompCytogen.v11i4.13962 (PMC5672327; doi:10.3897/CompCytogen.v11i4.13962)
Supplement: Supplementary material 1 — Voucher specimens [file comparative_cytogenetics-11-703-s001.docx]

# Supplementary material 1:

Voucher specimens: All analyzed specimens were deposited at Mammals Collection in the Instituto Nacional de Pesquisas da Amazônia (INPA); specimens are indicated by species, sampling sites, genus and collector number, followed by INPA collection number (in parentheses) when available.

**Karyotyped specimens at the figures: Figure 2:***Caluromys* *philander* (SISTAP-M-244, boxes: CAN 34, SISTAP-M-305); *Caluromys* *lanatus* (CTGA-M-701); *Marmosa* *demerarae* (RNL 46, boxes: MCA 27); *Marmosa* *demerarae* (RNL 46, boxes: MCA 27); *Marmosa murina* (RNL 69, boxes: CEF 18); *Marmosops pinheiroi* (INPA 5377, boxes: EE 192) (SISTAP-M-278, boxes: EE107, INPA 5408); *Metachirus nudicaudatus* (SISTAP-M-302; boxes: SISSIS-M-64): *Gracilinanaus emiliae* (SISTAP-M-243). **Figure 3:** *Glironia venusta* (BAC 80); b) *Monodelphis* aff. *adusta* (INPA 5388); c) *Monodelphis touan* (INPA 5404); d) *Monodelphis* sp. (CAN 44); e) *Didelphis marsupialis* (EE 249, boxes: EE174).

**Voucher specimens: *Glironia venusta*:** (BAC 80) - ***Caluromys philander*:** Tapajós River (male: SISTAP-M-297; SISTAP-M-305; SISTAP-M-318; SISTAP-M-382; female: SISTAP-M-244); Trombetas River (female: CTGA-M-652); Purus River (female: CAN 34); Manaus (female: MSN 01); (female: BAC 102) - ***Caluromys lanatus*:** Japurá River (female: CTGA-M-701) - ***Marmosops* sp.:** Aripuanã River (female: MCA 3; MCA 7; MCA 8; MCA 26; MCA 31; MCA 35; male: MCA 4; MCA 16; MCA 38; MCA 39); Jari River (female: TAG 3459; RNL 70); Juruá River (male: EE 107; EE 139; female: EE135); Cuieiras River (female: EE 198; EE 211; male: EE 192; EE 201; EE216) ***- Marmosops bishopi*:** Negro River (male: SISIS-M-127); Purus River (male: SISPUR-M-135; SISPUR-M-157; SISPUR-M-160; SISPUR-M-164; SISPUR-M-135; CAN 30; CAN 51; female: CAN 48) - ***Marmosops pinheiroi*:** Tapajós River (male: SISTAP-M-237; SISTAP-M-278; female: SISTAP-M-268; SISTAP-M-277) - ***Marmosops parvidens*:** Trombetas River (male: CTGA-M-501; CTGA-M-516; CTGA-M-531; CTGA-M-532; CTGA-M-551; CTGA-M-555; CTGA-M-581; CTGA-M-600; female: CTGA-M-533) - ***Marmosops impavidus*:** Purus River (male: SISPUR-M-149) - ***Marmosops* cf. *pakaraime*:** Japurá River (male: SISJAP-M-705) - ***Marmosa murina:*** Jari River (male: RNL 45); Uatumã River (male: CEF 4; CEF 8; CEF 18; CEF 27; CEF 28; CEF 32; female: CEF 16; CEF 34; CTGA-M-8; CTGA-M-22; CTGA-M-41;), Negro River (male: SISIS-M-57; SISIS-M-63); Trombetas River ( female: CTGA-M—519); Purus River (male: CAN 43); Japurá River (male: CTGA-M-708) - ***Marmosa murina:*** Aripuanã River (female: MCA12, Japurá River (male: SISJAP-M-764)- ***Gracilinanus emiliae:*** Tapajós River: (male: SISTAP- M-245; SISTAP- M-343; SISTAP- M-344; SISTAP- M-345) - ***Micoureus demerarae*:** Aripuanã River (female: MCA 27; MCA 36; MCA 58; MCA 65; male: MCA 21; MCA59); Jari River (female: RNL 31; RNL 48; male: RNL 30; MCA 32; MCA 46; MCA 49; MCA 58; MCA 61; MCA 64; MCA 66; MCA 67) Juruá River (female: EE136; male: EE 143); Manaus (female: EE 149: EE 150; EE 151; EE 154; EE 158; EE 159; EE 169; EE 222; EE 228; 229; EE 234; male: EE 157; EE 167; EE 170; EE 176; EE 189; EE 194; EE 196; EE 202; EE 215; EE 220; EE 235); Cuieiras River (female: EE 193; EE 219); Tapajós River (female: SISTAP-M-229; SISTAP-M-241; SISTAP-M-321; SISTAP-M-333; SISTAP-M-369; male: SISTAP-M-267; SISTAP-M-279; SISTAP-M-322); Trombetas River (female: CTGA-M-579; CTGA-M-590; CTGA-M-622; CTGA-M-667; CTGA-M-672; male: CTGA-M-535; CTGA-M-539; CTGA-M-557; CTGA-M-558; CTGA-M-572; CTGA-M-573; CTGA-M-578; CTGA-M-580; CTGA-M-613); Negro River (female: SISIS-M-85; SISIS-M-110; SISIS-M-117; SISIS-M-128; male SISIS-M- 86); Purus River (female: SISPUR-M-145; CAN 25; CAN 31; CAN 50: male: SISPUR-M-144; SISPUR-M-147; SISPUR-M-148) - ***Monodelphis* aff. *adusta:*** Madeira River (male: MCA 15) - ***Monodelphis touan:*** Jari River (male: TAG 2731; RNL 68) - ***Monodelphis* sp.:** Purus River: (male: CAN 44) - ***Monodelphis emiliae:*** Aripuanã River (female: MCA 31) - ***Metachirus nudicaudatus:*** Jari: River (RNL 47); Cuieiras River: (female: EE 200); Tapajós River (female: SISTAP-M-230; SISTAP-M-230; male: SISTAP-M-251; SISTAP-M-269); Trombetas River: (female: CTGA-M-655); Jatapú River: (female: CTGA-M-52; CTGA-M-58); Negro River: (female: SISIS-M-64; SISIS-M-78; male: SISIS-M-84; SISIS-M-116); Purus River: (male: CAN 33) - ***Didelphis marsupialis***: Jari River: (female: RNL 44; RNL 53; RNL 59; male: RNL 52; RNL 55; RNL 62; RNL 63); Manaus: (female EE 174; EE 197; EE 204; EE 224; EE 227; EE 246; EE 250; EE 155; EE 155; EE 173; EE 183; EE 190; EE 203; EE 205; EE 206; EE 223; EE 232; EE 233; EE 237; EE 247;EE 248; EE249; EE 190); Uatumã River (female: CEF 5; male: CEF 13); Trombetas River (female: CTGA-M-594; CTGA-M-606; male: CTGA-M-607); Purus River (male: SISPUR-M-185); Negro River (male: SISIS-M-73):Tapajós River (female: SISTAP-M-324; SISTAP-M-346; SISTAP–M-347;male: SISTAP-M-243); Japurá River: (male: CTGA-M-732).
